# Evaluation of electronic health record-integrated artificial intelligence chart review

**DOI:** 10.1038/s44401-025-00064-x

**Published:** 2026-01-28

**Authors:** Nicolas M. Kahl, Marshall J. Frieden, Zach R. Pope, Marlene M. Millen, Vaishal M. Tolia, Theodore C. Chan, Christopher A. Longhurst, Karandeep Singh, Alan X. You

**Affiliations:** 1 Department of Emergency Medicine, University of California, San Diego, CA USA; 2 Department of Family Medicine, University of California, San Diego, CA USA; 3 Division of Biomedical Informatics, Department of Medicine, University of California, San Diego, CA USA; 4 Division of Hospital Medicine, Department of Medicine, University of California, San Diego, CA USA; 5 Jacobs Center for Health Innovation, University of California, San Diego, CA USA

**Keywords:** Computational biology and bioinformatics, Health care, Mathematics and computing, Medical research

## Abstract

This study evaluates the quality of artificial intelligence (AI) clinical note summarization by analyzing physician qualitative feedback on a large language model (LLM) chart review tool integrated into the electronic health record (EHR). Physicians provided free-text feedback on AI-generated chart summaries, which physician informaticists analyzed using MAXQDA. Feedback from 10 physicians was collected on 147 AI-generated summaries. Positive feedback was common (*n* = 71), but users identified omissions (*n* = 46), confusing content (*n* = 20), token limitations (*n* = 27), hallucinations (*n* = 5), and bias (*n* = 1). Cohen’s Kappa was 0.64, indicating substantial reviewer agreement. Physician feedback on the tool revealed overall positive impressions, though omissions raised concerns about summary completeness. AI-assisted chart review technology is not infallible, but physicians found this tool acceptable for use in clinical workflows.

Artificial intelligence (AI) driven solutions have the potential to transform how physicians navigate and interpret patient charts. Reviewing prior notes within the electronic health record (EHR) is a fundamental aspect of clinical practice. This has become more difficult due to “note bloat,” with recent evidence showing that note length has increased by 60% over the past decade^1^. This phenomenon results in clinicians spending over 5 min to review a single patient chart based on a recent analysis of 100 million patient encounters with 155,000 physicians across 400 health systems^2^.

Recent advancements in natural language processing (NLP) and deep learning have resulted in large language models (LLMs) capable of processing and summarizing the heterogenous, unstructured, narrative free text that comprises medical notes^3^. Synthesizing patient clinical information from medical notes using LLMs has the potential to improve efficiency, decrease cognitive burden, lower the likelihood of missing key information, and reduce physician burnout^4,5^.

## Objective

While LLMs have demonstrated the ability to generate summaries of varying quality in controlled settings^5^, real-world clinician feedback on their accuracy, collected in a prospective manner, is lacking. With chart summarization now integrated directly into the EHR, characterizing the quality of AI-generated chart summaries is a pressing need. This study aims to address that gap by gathering and analyzing qualitative feedback from physicians using an EHR-integrated LLM chart review tool on real patient data.

## Outcomes of feedback analysis

Feedback was collected on a total of 208 AI note summaries. The vendor-required structured feedback showed in aggregate that 133 of 208 summaries were marked as positive and 75 of 208 summaries were marked as negative. Of the total 208 summaries, 147 contained free-text feedback, and 403 feedback codes were assigned by the two expert reviewers in aggregate. Following deduplication and harmonization to 11 unique qualitative codes, a second round of independent coding using the harmonized codes resulted in 221 unique instances of coded feedback. The harmonized codes were “positive feedback containing accurate/relevant information,” “too long,” “missing information,” “confusing information,” “hallucination or erroneous information,” “irrelevant information,” “redundant information,” “presence of bias,” “formatting recommendations,” “slow generation of summary,” and “technical limitations such as omissions due to token thresholds.” Interrater reliability revealed a Cohen’s Kappa of 0.64 with minimum code overlapping rate of 90% at the segment level, indicating substantial agreement between the two reviewers after the second round of independent coding.

Table 1 provides the assigned codes, counts, and exemplars. Many of the free-text comments reflected positive feedback on the AI-generated summaries (*n* = 71), but areas of improvement were also identified. While instances of hallucinations (*n* = 5) or bias (*n* = 1) were minimally reported, a substantially larger degree of feedback noted missing (*n* = 46) or confusing (*n* = 20) information. Issues related to input token limitations were also common (*n* = 27).

**Table 1:** Expert reviewer free-text feedback codes, counts, and examples

Expert Reviewer Categories	Count	Free-Text Feedback Examples
**Positive feedback (accurate/relevant)**	71	“Great summary of a complex patient.”
**Missing information**	46	“It would have been good if the summary also mentioned that she is getting worked up for a renal transplant - this was listed in one of the notes that was summarized but not in the summary.”
**Technical limitations / Token Thresholds**	27	“Token limit restricted summary to only 2 notes which was very limiting.”
**Formatting Recommendations**	23	“Included his long med list. Would be better if more than a few meds to not include in a prose form.”
**Confusing information**	20	“I’m not sure why it jumped from migraines to well-women exam?”
**Irrelevant information**	17	“The list of problems unrelated to her colonoscopy was irrelevant (e.g., allergic rhinitis, bunions, chicken pox, infertility).”
**Too Long**	6	“This ‘summary’ could have been reduced to 2 sentences instead of 5 lines (5 sentences) containing irrelevant information.”
**Hallucination / Erroneous Information**	5	“Cards visit assessment noted “Severe” AS - summary said moderate AS. Progress note reviewed prior echo reports, not noted in summary (or at least not commented on).”
**Slow Generation of Summary**	3	“Took 35 seconds to generate.”
**Redundant information**	2	“While all accurate, summary repeated an Adverse Medication reaction twice in one summary with different sources.”
**Presence of bias**	1	“Missed PMDD for which she had medication changes (so relevant for summary). Is it possible that LLM output is biased not to consider bothersome sxs of women?”

## Implications

Our study presents prospectively-collected qualitative evidence from 10 physicians providing 147 sets of free-text feedback on AI-generated chart summaries as part of standardized, EHR-integrated functionality that is currently being scaled nationally. The predominant finding was positive feedback, but inaccuracies were commonly reported. When information was inaccurate, we found that missing information and confusing information was more commonly reported than hallucinations. While hallucinations are a well-characterized problem with AI-generated note summaries evaluated previously^4,5^, our study suggests that errors of omission may represent a larger threat to the accuracy and usefulness of AI summarization tools than errors of commission (i.e., hallucinations). Together, these results indicate that the AI-enabled LLM note summarization tool was felt by end users to be clinically useful, while underscoring the need for improvements to prevent omission of pertinent clinical details.

One clinically meaningful example of hallucination that could impact patient safety occurred when the AI-generated summary substituted “severe” for “moderate” in characterizing a patient’s aortic stenosis. This substitution in disease severity highlights a potential risk that AI summarization of clinical notes could influence a physician toward inappropriate medical management. In the technology’s current state, the physician is responsible for verifying that the summary contains accurate information and would be liable for acting on erroneous information. There was also one instance of potential sex bias noted when the AI-generated summary did not include a description of symptoms related to premenstrual dysphoric disorder (PMDD) despite medication adjustments addressing the diagnosis. Such possible bias in AI summarization could lead to corresponding bias in a physician’s interpretation of patient medical history. Instances of omission were frequently noted by users when information was “listed in one of the notes that was summarized but not in the summary.” Despite having access to the needed input, the tool produced an incomplete summary. Our findings also demonstrated several instances in which the well-studied phenomenon of “note bloat” complicated attempts at chart review by exceeding the 8192 token threshold context window of the LLM chart review tool, thereby limiting the number of notes that could be summarized.

Our study did not directly compare the discrete feedback required by the vendor and the qualitative free-text feedback that we analyzed; however, our findings suggest that the qualitative feedback captured additional critiques that were not identified in the discrete feedback. In some instances, AI-generated summaries were rated as positive in the discrete feedback, but additional critical nuance was captured in the free-text feedback. This suggests that the open-ended format of free-text feedback allowed physicians to share detailed insights that may prove useful for the further development of AI note summarization tools.

AI-generated misinformation is an ongoing concern in the medical community. Hallucinations are particularly concerning, with one recent study finding that a widely used commercial LLM generated plausible but incorrect advice 55% of the time when asked about resuscitation of a non-breathing patient according to the 2021 Resuscitation Council United Kingdom Guidelines^6^. In addition to concerns about AI-generated misinformation, omission of information has also been documented in prior retrospective studies. In an evaluation of 100 LLM generated emergency department summaries, 47% were found to have omitted clinically relevant information^7^. Recently observed LLM error rates for medical text summarization of 450 clinical notes reported a hallucination rate of 1.47%, as compared to a 3.45% omission rate^8^. Efforts are ongoing to improve the accuracy of LLM generated information through improving model performance, in-context learning, and development of prompting frameworks^4^. Furthermore, iterative prompt engineering to tailor LLM tools to specific clinical contexts and use cases may also reduce the frequency of this issue. The AI-enabled LLM note summarization tool in this study utilized a single static prompt across all practice sites. This unified prompt strategy likely contributed to some component of the omissions reported, as emergency medicine physicians and primary care physicians prioritize different elements of a patient’s information in their clinical practice. Future iterations of AI-enabled LLM note summarization may be more likely to capture important patient information by using adaptive department-specific and use-case-specific prompts that tailor the workflow by specialty.

While AI note summarization has the potential to reduce some of the difficulties reviewing records affected by note bloat, unnecessarily long notes do still affect the ability of such tools to function. In this pilot study, the tool used was limited to only 8192 tokens, which is much lower than what is currently available in state-of-the-art large language models. We were constrained by the vendor-provided tool, which utilized Open AI’s GPT-4 version released in March 2023. Newer models have much larger context windows; for example, OpenAI’s GPT-5.1 model has a context window of 400,000 tokens, and Llama-4 Scout has a context window of 10 million tokens^9,10^. However, larger context windows do not necessarily equate to improved performance, as they require more time and financial resources to process and can sometimes introduce additional confusion in model output.

Our study is limited by deployment of a unified static prompt within a single multi-hospital health system, validation by a small number of physicians which may overrepresent outliers in feedback, and use of a small context window that may have resulted in omission due to lack of including necessary source notes. Despite these limitations, our study is important because it adds to the growing evidence that missing information remains the predominant challenge for AI note summarization tools. Practicing physicians may need workflows that enable access to the source data to allow for verification of AI-generated summaries. Missing information is harder to detect than hallucinations. Given the broad deployment of this tool within the EHR and its availability at a national scale, along with its vendor-provided standardized prompt that will produce similar results at other deployment sites, we believe our findings to be relevant to health systems across the country.

As these tools continue to be scaled nationally, our findings reinforce the need to conduct rigorous evaluation of assistive applications that help physicians responsibly integrate AI into clinical practice^11^. In light of missing and confusing information, physicians should consider AI-generated summaries of clinical notes as a starting point when time is short, but not as a replacement for manual chart review. AI-assisted chart review technologies are not infallible, but our findings suggest that physicians find this tool is overall acceptable for use to augment manual chart review in clinical workflows.

## Methods

### Large language model chart review tool

An EHR-integrated LLM chart review tool that performs chart summarization at UC San Diego Health was implemented with Epic Systems (Verona, WI). This tool selects a subset of clinical notes (hospital discharge summaries, emergency department physician notes, and outpatient clinic physician notes) and produces a focused summary of their contents. The AI note summarization tool was integrated within the EHR and used OpenAI GPT-4 via a single, static, standardized prompt to generate note summaries. The GPT-4 version utilized was the original version released in March 2023. This iteration of Epic’s integrated note summarization tool was targeted at ambulatory and ED settings. The tool was limited to 8192 tokens total for input and output, with tokens defined as the units used by AI systems to measure text length. Users accessed the tool within the EHR interface and were able to select notes for summarization directly from the list of clinical notes stored in the EHR.

### Data collection

We prospectively collected user feedback from August to September 2024 as part of a predeployment quality-improvement safety assessment of the tool within a test environment using recent patient records. Users included 10 physicians total: 6 from emergency medicine and 4 from outpatient primary care. The physicians had prior experience using LLM software, but not using the specific tool in this pilot study. Feedback was collected via an interface embedded within the EHR. In addition to supplying required structured discrete feedback to the vendor, users at our institution were encouraged to enter free-text feedback during the evaluation period.

### Data analysis

The free-text feedback was qualitatively analyzed by two expert clinical informaticist reviewers in December 2024. Extracted raw free-text feedback was evaluated for any protected health information (PHI), and any PHI was removed prior to evaluation. Then, the raw feedback was reviewed to identify common themes. Each free-text feedback response was subsequently coded with custom codes by each of the two reviewers using MAXQDA software (VERBI Software, Berlin, Germany). After an initial round of coding by each reviewer, code sets were harmonized to 11 total codes, and each reviewer then performed a second round of coding and independently reassigned these harmonized codes to all applicable segments of the free-text feedback.

Agreement between the two reviewers was determined as assignment of the same code to a segment of free-text feedback with at least 90% character overlap of the text spans identified by the two reviewers. Quantitative analysis of interrater reliability was determined using MAXQDA software to generate Cohen’s Kappa values with prespecified segment overlap after the second round of independent coding.

## Supplementary information

Supplemental information

## Data Availability

Due to the AI-generated note summaries and physician feedback containing protected health information, the data used in this study cannot be publicly shared. Table 1 contains de-identified exemplars of physician feedback.

## Code availability

The full software code for the AI note summarization tool cannot be disclosed, as it is proprietary to Epic Systems. The large language model is available from OpenAI.
