# Supplementary material for: Evaluation of electronic health record-integrated artificial intelligence chart review
Source: Npj Health Syst. 2026 Jan 28;3:6. doi: 10.1038/s44401-025-00064-x (PMC13354172; doi:10.1038/s44401-025-00064-x)

Supplemental Information

Large language model utilized: <https://platform.openai.com/docs/models/gpt-4>

Single, static, standardized prompt: Prompt text removed at the author’s request, owing to the lack of necessary permissions from Epic Systems.

Screenshot of user interface:


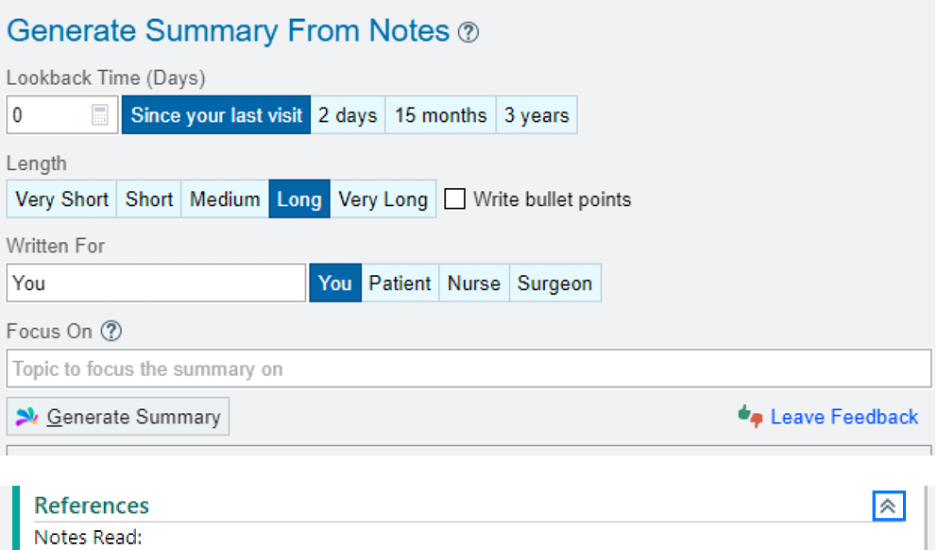

Supplement: Supplementary file 1 — Supplemental information [file 44401_2025_64_MOESM1_ESM.docx]
